# Vaccination Failures in Pigs—The Impact of Chosen Factors on the Immunisation Efficacy

**DOI:** 10.3390/vaccines11020230

**Published:** 2023-01-19

**Authors:** Agata Augustyniak, Małgorzata Pomorska-Mól

**Affiliations:** Department of Preclinical Sciences and Infectious Diseases, Poznan University of Life Sciences, Wolynska 35, 60-637 Poznan, Poland

**Keywords:** pigs, vaccines, vaccination, efficacy, interference

## Abstract

Infectious diseases that often lead to economic losses still pose a severe problem in the pig production sector. Because of increasing restrictions on antibiotic usage, vaccines may become one of the major approaches to controlling infectious diseases; much research has proved that they could be very efficient. Nevertheless, during their life, pigs are exposed to various factors that can interfere with vaccination efficacy. Therefore, in the present paper, we reviewed the influence of chosen factors on the pig immunisation process, such as stress, faecal microbiota, host genetics, the presence of MDAs, infections with immunosuppressive pathogens, and treatment with antibiotics and mycotoxins. Many of them turned out to have an adverse impact on vaccine efficacy.

## 1. Introduction

Infectious diseases still pose one of the greatest threats to pig production. In addition, intensive husbandry may increase the susceptibility of pigs to some respiratory and intestinal infections [1]. Many of them can lead to reduced production parameters and increased animal collapses, resulting in economic losses. It is well known that pathogens are responsible for most of the economic losses in the livestock sector [2,3]. 

These days, one of the most important approaches to controlling infectious diseases is vaccination. Considering the increasing trends of restricted antibiotic use to inhibit antimicrobial resistance, proper vaccination protocols may become even more important; vaccines are among the five most effective alternatives to antibiotics [4]. Numerous vaccines were proven to be efficient in protecting pigs against infections with various pathogens, bacterial as well as viral, or against infections’ adverse effects. Nevertheless, in some cases, vaccines can be less effective than expected or even completely inefficient. Even using the same vaccine, immunisation efficacy can differ among particular individuals [1,3]. It may result from an abundance of factors that can influence vaccination [5]. Approximately 40 factors that can impact this process were determined in humans, including intrinsic host factors (e.g., age or genetic), perinatal host factors (e.g., maternal infections during gestation, presence of maternal antibodies), extrinsic factors (e.g., infections, parasites, microbiota), behavioural factors (acute and chronic psychological stress, sleep), nutritional factors, environmental factors or vaccine- and administration-related factors [5]. 

Vaccination efficacy can be determined based on obtained final results, such as disease prevention, reduction in the clinical course of the disease or immune response [6]. Heininger et al. (2011) observed some issues with the definition of vaccination failure. They, therefore, divided the causes of this phenomenon into two main groups: vaccine failure and failure to vaccinate. Vaccine failures were further divided into host-related and vaccine-related factors. In pigs, host-related factors involve, e.g., immunodeficiency, suboptimal immune response, immaturity of the immune system, inadequate health status, waning immunity, some immunological interference such as maternally derived immunity (MDI) or some incubating infections [7]. The second mentioned group includes such issues as low vaccine potency, the imperfect antigenic match between field and vaccine (strain, serotypes, genotypes or antigenic variants), interference with other co-administrated vaccines or some manufacturing problems [6,7]. Within the failure of the vaccine, two subgroups were distinguished [6]. The first one was related to incorrect usages, such as the wrong vaccine dose or administration route, lack of booster, inappropriate storage conditions or vaccine use beyond the expiry date [6,7]. The second one included various program-associated problems such as vaccine availability.

## 2. Stress

During life, pigs are exposed to many stressors, such as weaning, social integration (crowding/mixing/isolation), transport, food deprivation, light/dark cycles, novel environments, diseases or temperature issues [8]. It has been documented that cortisol, as well as other neuroendocrine components of the stress reaction, can influence the immune system [9]; for example, pigs with elevated cortisol levels exhibit inhibited proliferation of lymphocytes to mitogens [10]. Based on these findings, it can be thus assumed that various stressors may impact the efficacy of the porcine vaccination. However, the available data on this subject could be more abundant. The influence of mixing unfamiliar pigs on the specific immune response against the pseudorabies virus (PRV) vaccine has been investigated previously [9]. The mixing increased agonistic behaviour, cortisol concentration in saliva and catecholamine excretion in urine independent of the pigs’ gender [9]. However, in mixed gilts, no differences in comparison to control gilts were observed; meanwhile, mixed barrows displayed inhibited post-vaccinal immune response and exacerbated clinical signs following PRV exposure contrary to control barrows [9]. Following vaccine administration, restimulated lymphocyte proliferation, immunoglobulin (Ig) M, interferon (IFN) γ, and interleukin (IL) 10 responses were documented to be decreased in the group of mixed barrows. Moreover, mixed dominants seemed to be more severely affected than subordinates [9]. Thus, it can be assumed that mixing unfamiliar pigs can suppress the immune response to a viral vaccine and further adversely influence protection against infection [9]. Another team of researchers evaluated the efficacy of *Mycoplasma hyopneumoniae* vaccination at one of the most stressful moments of the pigs’ lives, that is, the weaning process [11]. In the mentioned study, two groups of swine were vaccinated with one dose of *Mycoplasma hyopneumoniae* bacterin vaccine: one three days before weaning and the second one on the day of weaning and subsequently, four weeks later were inoculated with *Mycoplasma hyopneumoniae*. In the results, both vaccinated groups exhibited a decrease in macroscopic and histopathological lung lesions and a lower number of *Mycoplasma hyopneumoniae* in the broncho-alveolar lavage fluid compared to the non-vaccinated control group. Statistically significant differences between the two trial groups were observed only in the matter of microscopic lung lesions, which were lower in the group of pigs that obtained the vaccine three days before weaning; in this group, pigs displayed a lower average lympho-histiocytic infiltration score and the highest average percentage of air in the lung tissue when compared to the two remaining groups. The authors concluded that based on the obtained result, it could not be firmly determined whether weaning influenced vaccination efficacy, and further research is needed [11].

## 3. Faecal Microbiota

One of the most recent reports on factors likely to influence vaccine efficacy concerns faecal microbiota. Nevertheless, these data are still scarce. It is well known that the gut microbiota plays an essential role in providing health to its host; it participates in forming mucosal and systemic immunity [12]. Borey et al. (2021) showed that among piglets vaccinated against the influenza A virus (IAV), a higher post-vaccinal response was observed in the group characterised by richer microbiota [13]. The authors determined several operational taxonomic units, present at the early stages of piglets’ life, that might positively as well as negatively impact post-vaccinal immunity; the better immune response was linked with operational taxonomic units included in the genus *Prevotella* and family *Muribaculaceae*; meanwhile, worse responses were linked with those included in the genera *Helicobacter* or *Bacteroides*. Therefore, an improvement in vaccination efficacy may be achieved by some farm and breeding practices that lead to the enrichment of the gut microbiota [13]. Interesting results were also obtained in the study in which the course of infection with two different strains of ASFV was compared between two groups of pigs: conventional and specific-pathogen-free (SPF); the higher baseline innate immunity activity present in the group of conventional pigs allowed a reduction in initial virus replication, but during infection with attenuated ASFV strains it led to immunopathological cytokine responses as well as delayed lymphocyte proliferation [14]. Severe clinical signs, such as viremia and the production of pro-inflammatory cytokines as a consequence of inoculation with highly virulent ASFV, were observed earlier in the SPF pigs, which were marked by lower white blood cell counts and lower basal inflammatory and antiviral transcriptomic profiles at the steady state contrary to the conventional pigs. On the other hand, after inoculation with an attenuated field ASFV isolate, the SPF pigs were characterised by a much milder clinical course of infection that ended with recovery; meanwhile, in conventional pigs, a severe form of the disease with high mortality was noted [14]. Differences among these two groups were also observed in the matter of produced cytokines: the SPF pigs produced more anti-inflammatory cytokines, contrary to the conventional pigs, which were characterised by a higher production of inflammatory cytokines [14]. The results of this study may be crucial for the development of an effective ASF vaccine.

## 4. Pigs’ Genetics

Host genetics is one factor that influences vaccination effectiveness; differences in this issue may result from particular individuals’ genomic diversity [1,15]. For example, it is well known that in humans, single nucleotide polymorphisms (SNPs) in human leukocyte antigen (HLA) class I and II, as well as cytokine receptor or innate immune components genes, may result in some differences in immune reaction to vaccines against different pathogens [15]. The first reports about the importance of genetics in pig vaccination came in 1984 [16]. In the mentioned study, a group of 518 four-week-old pigs of multiple breeds (Chester White, Duroc, Hampshire, Landrace, Yorkshire breeds) were vaccinated against pseudorabies. Four weeks later, antibody titres were assessed using a microtitration serum-neutralisation test. This experiment showed significant differences in antibody response among individual pig breeds; Duroc and Landrace pigs exhibited the lowest antibody response level [16]. Knowledge about high polymorphism in swine leukocyte antigen (SLA) as well as in numerous porcine toll-like receptor (TLR)—TLR 1, TLR 2, TLR 4, TLR 5 and TLR 6—genes has encouraged a team of researchers to conduct a study on whether these factors influence the effectiveness of antibody response after vaccination [1]. A total of 191 Duroc pigs maintained under specific-pathogen-free conditions were immunised with *Erysipelothrix rhusiopathiae,* and *Actinobacillus peluropneumoniae* (APP) serotypes 1, 2 and 5. The results of this experiment exhibited a relationship between different genotypes of SLA class II and TLR genes and various types of vaccination antibody response. A decrease in antibody response to *Erysipelothrix rhusiopathiae* immunisation was observed in pigs with specific haplotypes in SLA class II. TLR 5 was strongly correlated with antibody response to APP2 and APP5; however, such a correlation was not observed in the case of APP1. Pigs with specific haplotypes in TLR5 displayed an enhanced response to APP2 and APP5 vaccination [1]. Pigs’ genetics also plays an important role in developing an immune response after influenza A virus (IAV) vaccination. The result of the study performed on 103 crossbreed piglets vaccinated with inactivated H1N1pdm 2009 virus showed seven genomic regions related to post-vaccination immune response [3]. Using a haemagglutination inhibition (HI) assay, three markers with unknown locations and three on chromosomes SSCX, SSC14 and SSC18 were indicated as related to immune response. The following four genomic regions, found on chromosomes SSC12, SSC1, SSC7 and SSC15, were associated with an immune response when the association was tested with an ELISA assay [3]. Host genetic and blood transcriptomics were proved to cause high differences among individuals in antibody levels following *Mycoplasma hyopneumoniae* vaccinations, which were affected by host genetics as well as blood transcriptomics [15]. In the quoted study, 182 Large White pigs were immunised twice (at weaning and 21 days later) against *Mycoplasma hyopneumoniae*. Levels of *Mycoplasma hyopneumoniae* antibodies were measured (at 0, 21, 28, 35 and 118 days post-vaccination) to asses vaccine response. In the result, a variability of *Mycoplasma hyopneumoniae* antibody levels among particular pigs at all time points was observed; females exhibited higher antibody titres compared to males at the latest time point. This variability further corresponded with heritabilities that ranged between 0.46 and 0.57. Moreover, the authors detected two genomic regions associated with observed variability in *Mycoplasma hyopneumoniae* antibody titre at 21 days post-vaccination. These genes were located on chromosome 1 and chromosome 4. This paper also confirmed that the pre-vaccination blood transcriptome co-varies with antibody response [15]. To sum up, these findings may indicate the importance of pigs’ genetics in the immune response to vaccination.

## 5. Presence of MDAs

Vaccines can be administrated to sows, piglets or both. However, the immunisation of the offspring from vaccinated or previously infected sows is the next factor that influences vaccination effectiveness due to the possibility of interference of MDI with vaccines [17]. It is well known that maternally derived antibodies (MDAs) during vaccination may lead to a reduction in or complete inhibition of the immune response against inactivated but also live vaccines [18]. Piglets are considered to be born immunocompetent; however, they are genuinely immunologically defenceless. Porcine placenta belongs to the epitheliochorial type; it effectively precludes the transfer of immunoglobulins and immune cells [19]. Survivability at the early stages of extrauterine life is thus provided by MDI from first, colostrum and, later, milk. From an immunological point of view, the most important colostrum component is immunoglobulins (IgG, IgA and IgM). However, other factors such as cytokines or immune cells (epithelial cells, macrophages, NK cells, T- and B-cells) also play a significant role in protecting the neonate [19]. MDAs are efficient only in protecting piglets against pathogens their sow has faced due to natural infection or vaccination. Various factors determine the amount of MDI transferred from the sow to its offspring [19,20]. The ideal moment for conducting vaccination in piglets is when MDI declines to the level that enables the development of long-term cellular and humoral immunity but is still sufficient to protect against infection [17]. We can distinguish two terms that are used to determine the presence of MDAs. The first is called the rate of MDA decay, also known as “half-life”, and refers to the time required for a 50% decrease in MDA concentration [21]. The second indicates the piglet’s age at which the MDA’s level becomes undetectable [21]. The duration and level of MDI, however, are not equal even among piglets in the same group. It depends on the immunological status of the sows as well as the piglet’s colostrum intake [21,22]. These differences may pose a serious problem in determining optimal vaccination moments. MDAs (especially high titres) may suppress the development of active humoral response; the data relevant to cell-mediated immune (CMI) responses are inconsistent [17]. Some studies suggest that MDAs do not inhibit CMI response after vaccination [23], while others indicate that MDAs interfere with it [17,24]. Moreover, some studies suggest that despite MDAs adversely influencing seroconversion, they do not interfere with vaccination effectiveness. Therefore, in the next part of this paragraph, we reviewed the available literature regarding specific MDA interference with vaccines against various infections (Table 1).

### 5.1. Classical Swine Fever Virus

Classical swine fever (CSF), also known as hog cholera, is caused by the classical swine fever virus (CSFV) and still poses a serious problem in many countries. In endemic CSF areas, the prevention and control of this disease depend mainly on vaccinations. The duration of the MDA against CSFV was assessed as being longer than seven weeks, with a steady decline in antibody titre until ten weeks of age [25]. The mean half-life of colostral antibodies was assessed as 7.9 days [25]. It was also demonstrated that MDA-positive, control piglets born to sows vaccinated with one dose of C-strain CSFV displayed reduced mortality to 30% following CSFV challenge at the age of seven weeks and to 50% when the CSFV exposure took place at the age of ten weeks, in comparison to piglets devoid of MDAs [26]. Several studies examined the impact of MDAs on the efficacy of vaccination against CSFV. One of them demonstrated that the vaccination of neonates before first suckling was efficient in protecting against severe disease following challenge with CSFV, and the administration of a vaccine after colostrum intake was inefficient; 82% of piglets vaccinated after first suckling did not survive the subsequent CSFV challenge. The remaining piglets survived but exhibited clinical signs of the disease [25]. All pigs vaccinated at the age of seven weeks resisted the virus exposure [25]. These findings indicate that MDAs may interfere with CSFV vaccine efficacy. This statement can be supported by a subsequent study in which pigs vaccinated with lapinized Chinese-strain CSF vaccine in the presence of high MDA titres displayed an inhibited cellular as well as antibody response [27]. Moreover, it was possible to isolate CSFV from approximately 50% of these pigs following virus exposure. In contrast, it was observed that piglets vaccinated with low titres of passive antibodies exhibited a significantly higher number of CSFV-specific IFN-γ-secreting cells and were fully protected during the CSFV challenge [27]. Marker vaccines that may enable the differentiation of infected from vaccinated animals (DIVA) are considered a helpful tool in eradicating CSF in endemic areas; thus, knowledge about the possibility of interference of MDAs with particular CSFV marker vaccines seems to be substantial. One example of such a marker vaccine against CSFV is the E2 subunit vaccine [28]. The immunisation of two-week-old, MDA-positive piglets with the E2 subunit vaccine led to reduced levels of vaccine-induced antibodies three and six months following vaccination. Moreover, six months later, these pigs also exhibited less effective protection against CSFV transmission [28]. Three studies evaluated the efficacy of another marker vaccine, CP7_E2alf, in the presence of MDAs [26,61,62]. First of them, evaluated whether MDAs, induced by the immunisation of sows with the C-strain vaccine, influenced vaccination efficacy with the CP7_E2alf vaccine in their progeny. MDA-positive piglets were vaccinated at five or eight weeks and subsequently, two weeks later, were exposed to highly virulent CSFV. As a result, no severe clinical signs, pathological lesions, or mortality were observed in the vaccinated group; moreover, in vaccinated piglets at the time of the virus challenge, an increase in the ELISA antibody titre was observed [26]. No major differences in vaccine efficacy were observed between piglets vaccinated at five and eight weeks of age [26]. In the subsequent study, piglets that were born to CP7_E2alf vaccinated sows and subsequently immunised with the same marker vaccine were protected against severe clinical signs of CSF [61]. Despite that shedding and transmission of the virus were noted, transmission in the vaccinated piglets was significantly lower, contrary to non-vaccinated piglets [61]. Nevertheless, the authors observed that the CP7_E2alf vaccination efficacy in the face of MDAs was slightly decreased compared to the vaccination of pigs devoid of MDAs [61]. Based on this study, it could be pointed out that the route of administration may influence the efficacy of CP7_E2alf vaccination [62]. It has also been found that the presence of MDAs had an adverse impact on vaccination effectiveness; however, the degree of this negative effect depended on the vaccine’s administration route [62]. Intramuscular administration was a more efficacious route than oral application; in the group of MDA-positive piglets that obtained the vaccine intramuscularly, the prevention of mortality, reduction of clinical signs and virus level in the blood as well as increasing antibody levels were noted [62]. Thus, it can be assumed that the CP7_E2alf vaccine can effectively control and eradicate CSF on a population level, even in the presence of MDAs.

### 5.2. Foot and Mouth Disease Virus

Foot and mouth disease (FMD) is a highly contagious disease of cloven-hoofed animals caused by the foot and mouth disease virus (FMDV); it also poses a significant threat to pigs. In several countries with FMD-free status, vaccination against FMDV is forbidden. However, in many other countries, FMD outbreaks pose a serious problem, and vaccination is an effective approach to disease control [63]. Thus, knowledge about efficient vaccination protocols still plays an important role. It has been established that the protection of the MDA against FMD only lasts a while, and 50 days is enough for the MDA to decrease from positive to negative values [63]. Interestingly, the duration of MDAs against particular serotypes is different [22]. It has been reported that MDAs against FMDV may interfere with vaccines [29,30]. The interference of MDAs against FMDV with vaccine occurred in one-, two-, as well as in four-week-old piglets and even partially in eight-week-old piglets. Moreover, the vaccination of MDA-positive piglets at this age did not confer any protection against experimental infection at six to seven months of life [29]. Liao et al. (2003) demonstrated that piglets born to immunised sows and vaccinated against FMDV at the age of 8 weeks had significantly higher mean serum-neutralisation (SN) antibody titre at the age of 24 weeks in comparison to piglets that were vaccinated at the age of 2 or 4 weeks. Moreover, after the challenge with FMDV, all piglets vaccinated at eight weeks of age were protected against infection. Meanwhile, piglets immunised at the age of four weeks exhibited clinical signs of FMD [30]. The authors also revealed that the difference between the SN titres before and two weeks post-vaccine administration was adversely associated with the SN titres before vaccination; an increase in SN titre at two weeks post-vaccination could be achieved only when MDAs decreased below the level of 2.23 (derived from the regression line) at the time of vaccination [30]. In the study of Dekker et al. (2016), MDA-positive piglets vaccinated at seven or nine weeks of age exhibited a similar reaction to immunisation as MDA-negative piglets vaccinated at the age of three weeks. Thus, vaccination was considered sufficient. Interestingly, in the quoted study, contrary to another report [29], MDA-positive piglets immunised at the age of three weeks developed a post-vaccinal reaction [22]. This finding, collectively with the study of Liao et al. (2003), may suggest that piglets can develop the post-vaccine response against FMD in the presence of MDAs [22,30]. Nevertheless, Dekker et al. (2016) displayed that higher levels of MDAs during piglet immunisation resulted in poorer post-vaccination response [22]. An optimal moment for FMD vaccination was determined as eight weeks of age [30,64,65]; it seems to be an adequate moment not only due to developing sufficient protection but it also enables avoiding an immunity gap [65]. To provide a good level of protection in endemic areas, two doses of the FMDV vaccine are recommended. In the study of Lee et al. (2013), the double vaccination of piglets (at both 8 and 12 weeks of age) resulted in better protection in comparison to single vaccination at the age of 8 weeks [64]. Nevertheless, previous research by Liao et al. (2003) showed that high levels of serum-neutralisation (SN) titres, as well as complete protection at 24 weeks of age, may be provided by a single dose of high-potency vaccine at 8 weeks of age [30]. Kim et al. (2019) indicated that the age of eight weeks could be considered an optimal moment for one-dose FMDV vaccination when the booster is unavailable. In this case, the single vaccination of eight-week-old piglets led to better seroprevalence than in older piglets; however, those piglets did not achieve sufficient antibody levels for FMDV protection [65].

### 5.3. Pseudorabies Virus (Aujeszky’s Disease)

The MDAs against pseudorabies virus (PRV) obtained from vaccinated sows may disturb or even completely inhibit the development of post-vaccination active humoral response [17]. The duration of the MDAs in piglets born to naturally or experimentally infected sows was assessed in a study from 1988 [40]. It proved that the levels of serum and colostrum immunoglobulins were associated with the clinical course of the disease. The offspring of naturally as well as experimentally infected sows with highly virulent PRV strains received a higher amount of specific antibodies. These MDAs were persistent much longer than those obtained from experimentally infected sows that presented only mild signs of disease; in this case, piglets were devoid of specific MDAs after three weeks of life [40]. The duration of MDAs was assessed as 10–11 weeks of age [17]. Pomorska-Mól et al. (2010) observed that piglets vaccinated once at eight weeks of age did not develop any humoral response to the vaccine antigen. Moreover, piglets that were immunised twice—at one and eight weeks of life—exhibited a similar response to piglets vaccinated once at eight weeks of life [17]. Pigs vaccinated at 1 and 12 weeks of age displayed a higher mean ELISA S/N ratio than those revaccinated at 8 weeks. An active humoral response was observed only in the first-mentioned groups [17]. Another study compared the humoral response observed after PRV vaccination in different schedules [41]. According to it, the highest level of antibodies measured at the age of 18 weeks was noted in pigs that obtained the vaccine three times, at 8, 12, and 16 weeks of age; the administration of the last dose at the age of 14 weeks had limited effectiveness, which is in contrast to study of Pomorska-Mól et al. (2010), in which the highest and long-term humoral response was observed in pigs vaccinated twice at 10 and 14 weeks of life [17,41]. De Smet et al. (1994) observed that the percentage of seropositive pigs immunised against PRV once, at the age of 10 weeks, was two to three times lower compared to piglets that obtained two vaccine doses at 10 and 14 weeks of age [42]. Moreover, multiple vaccinations of sows with the attenuated Bartha strain resulted in a weaker induction of antibodies compared to the subunit vaccine [42]. The authors compared the active immunity following PRV vaccination at the end of the fattening period. According to this, the percentage of seropositive pigs at the end of fattening was significantly higher in piglets born to sows vaccinated with an attenuated strain compared to those from sows immunised with a gE-deleted subunit vaccine or PRV infection and thus with low MDA titres [42]. Collectively, the results of these studies indicate that a high level of MDAs may disenable the production of an active humoral response after PRV immunisation [17]. The presence of MDAs did not inhibit the early priming of T-cells; they were successfully primed even in piglets vaccinated at the age of one week [17]. Nevertheless, maintaining long-term cellular immunity demands the application of a minimum of one booster [17]. Based on the above result, the optimal moment for PRV vaccination was assessed as follows: the first dose at 10 weeks of life and then the booster at 14 weeks of life [17]. This seems to be an adequate time for vaccination. Due to the MDA level, it is still sufficient to protect piglets, but it does not interfere with developing their own immunity in reaction to immunisation [17]. Fischer et al. (2003) suggested that DNA vaccines may solve the problems associated with high MDI levels in conventional vaccination strategies [66]. The immunisation of eight-week-old piglets with a DNA vaccine led to the development of an active serological response to PRV. Repeated vaccination at the age of 11 weeks resulted in a clear anamnestic response. Moreover, the immunisation of 5–6-day-old piglets that displayed high titres of MDAs selectively suppressed a serological response; however, it did not affect the establishment of potent memory responses [66].

### 5.4. Parvovirus

It has been documented that the MDA against porcine parvovirus (PPV) can be present in the piglets’ for a long time [38]. Wrathall et al. (1987) conducted a study in which serum samples from a total number of 74 piglets from three successive litters of two sows vaccinated against PPV were tested for haemagglutination-inhibiting (HI) antibodies at different intervals of time [38]. This research showed that almost half of the pigs maintained positive MDA titres until six months of age. Moreover, traces of antibodies were still present in a few pigs at the age of nine months. This poses a risk of disability to developing an active immune response to PPV vaccination even at the onset of breeding. Three groups of piglets immunised against PPV at 70, 130 or 190 days of life developed a strong and long-lasting antibody response, even though they still exhibited moderate to high MDA titres [38]. Paul and Mengeling (1986) assessed the development of immune response after vaccine administration in pigs with various HI titres for PPV [39]. The reaction to the PPV vaccine in pigs with a low HI titre (1:5) was similar to the reaction in seronegative pigs. In the case of pigs with medium HI titres (1:10 or 1:20), the titre growth was not observed until the second dose of vaccine was administrated. Pigs with high HI titres (1:40 or 1:80) did not respond to vaccination [39]. These results suggest that low MDA levels do not compromise PPV vaccination effectiveness [39].

### 5.5. Porcine Reproductive and Respiratory Syndrome Virus

The vaccination of sows with modified live virus (MLV) vaccines provides piglets with high titres of the MDAs against porcine reproductive and respiratory syndrome virus (PRRSV) [24]. These MDAs are present in piglets until 2–11 weeks of age [24,43]. They remain relatively high until the age of four weeks; nevertheless, by that time, a gradual decrease in its level can be observed [44]. MDA titres reached the lowest levels at 6–8 weeks of age; in one of the examined groups, a decline in the percentage of piglets that exhibited positive SN titres from 80% at the age of four weeks to 7.9% at the age of six weeks was observed [44]. Fablet et al. (2016) evaluated whether MDAs affect post-vaccinal humoral or cellular immune response [24]. In this experiment, piglets with both high and low MDA levels were vaccinated at the age of three weeks. As a result, no vaccine viremia was detected during the first four weeks post-vaccination in the serum of piglets vaccinated with high MDA titres; however, 32% and 6% of these piglets were PCR-positive at 8 and 14 weeks after vaccination, respectively [24]. Neutralising antibodies in this group of piglets were detected only at 14 weeks of age. In the group of piglets that were vaccinated with lower MDA titres, PRRSV vaccine strains, as well as neutralising antibodies, were detected earlier. Moreover, piglets vaccinated with high MDA titres were characterised by a significantly lower number of PRRSV-specific IFN-γ-secreting cells at two and four weeks post-vaccination compared to piglets vaccinated with low MDA titres [24]. Additionally, Renson et al. (2019) proved that the vaccination of piglets in the presence of the MDAs against PRRSV might influence the post-vaccination immune response and vaccine strain replication [45]. At two weeks post-vaccination, vaccine strains were present only in 6% of piglets with high MDA levels; in contrast, in piglets vaccinated with low MDA titres, vaccine strains were detected in 69% [45]. Five weeks following vaccination, only 44% of high-MDA piglets seroconverted; meanwhile, in the low-MDA group, 94% of piglets did [45]. Moreover, in the group of piglets immunised in the presence of high MDA titres, viremia caused by the challenge was not significantly reduced compared to non-vaccinated piglets [45]. All of this may indicate that MDAs can adversely influence the post-vaccinal humoral and cellular response against PRRSV.

### 5.6. Porcine Circovirus Type 2

Numerous research was conducted on the interference of MDAs with piglets’ PCV-2 vaccination. Depending on its initial concentration, the MDA against PCV-2 may decline at different ages, between 2–15 weeks of life [31]. Numerous studies have shown that high levels of the MDA against PCV-2 can interfere with an active seroconversion [31,32,33,34,35,36,37]. Interestingly, despite this interference, PCV-2 vaccines can be effective; some researchers documented that PCV-2 vaccines administrated to piglets with the MDA were efficient in the reduction of PCV-2 viremia, virus shedding as well as the viral load in tissues or prevention of macroscopic and microscopic lesions [31,32,33,36]. Some studies also indicated that the MDA did not seem to interfere with the effect of the PCV-2 vaccine on average daily weight gain (ADWG) [35,36,37,67]. Nevertheless, Feng et al. (2016) mentioned that high MDA titres during vaccination might interfere with some production parameters [37]. One study pointed out that PCV-2 subunit vaccines can induce cellular immunity, and that response was not affected even in the presence of high MDA titres [37]; it can be thus suspect that a.o. the cell-mediated immune response may contribute to protecting the vaccinated MDA-positive piglets [35]. Haake et al. (2013) observed that the immunisation of piglets against PCV-2 at one week of age might have caused decreased production parameters and significantly higher viremia following PCV-2 challenge in comparison to vaccination at later stages of life (at three weeks of age) independently of antibody titre [35]. As the MDA levels were comparable in both groups of piglets (three-week-old piglets, however, were characterised by lower antibody levels in comparison to younger ones), the authors suggested that other age-related factors, but not MDAs, may interfere with the vaccine at the age of one week [35].

### 5.7. Swine Influenza Virus

The data concerning the duration of MDI against swine influenza A virus (SIV) was described in several studies. In Kitikoon et al. (2005), the MDA against SIV was present for up to 10 weeks of life [46]. This is consistent with another study, where the time of MDA decline was assessed as 10 weeks [47]. Markowska-Daniel et al. (2011) established the duration of the MDA against H3N2 SIV for 9–10 weeks; the MDA against H1N1 SIV remained positive up to 13–14 weeks [18]. In the experiment by Rajao et al. (2016), the MDA achieved a low level at 13 weeks of age, and at 16 weeks of age, it was below the detection level; some pigs, however, still had a detectable MDA at that time [48]. It is worth mentioning that the quoted studies had an experimental character. Rose et al. (2013) performed research in field conditions; MDA titres declined over time until 70 days (10 weeks) of age in two farms and until 50 days (~7 weeks) in one farm [49]. Several studies observed an interference of MDAs with vaccines against SIV [18,46,48]. In Markowska-Daniel et al. (2011), five groups of MDA-positive piglets were immunised twice, but each group was at a different age [18]. The administration of the first dose of the vaccine did not result in any seroconversion in any of the groups. The production of antibodies was observed after the second dose of the vaccine, even in the presence of MDAs; nevertheless, MDAs caused decreased antibody response [18]. The results of this study are partially consistent with an earlier one, published by Kitikoon et al. (2006), in which piglets vaccinated in the presence of MDAs did not exhibit any growth of haemagglutination inhibition (HI) antibody titres approximately two weeks after the administration of the first vaccine dose [46]. In comparison, in MDA-negative piglets that were immunised simultaneously, an increase in HI titres following vaccination was observed [46]. It was also observed that MDAs could inhibit the induction of post-vaccination SIV-specific memory T-cells [46]. Moreover, vaccination in the presence of MDAs may decrease vaccine effectiveness due to prolonged fever, prolonged clinical signs and more severe pneumonia [46]. The authors imply that these findings may question the vaccination of sows to increase MDA titres in piglets since it was also documented that the immunisation of piglets provides better protection against SIV than MDAs [46]. Additionally, Rajao et al. (2016) showed that in MDA-positive piglets immunised with the same vaccine as their sows, no increase in neutralising antibody level was observed against the vaccine strain [48]. Piglets vaccinated with another quadrivalent vaccine exhibited a detectable antibody response only against this vaccine component, which did not cross-react with their specific MDAs; nevertheless, the titres observed in this group were still lower when compared to MDA-negative controls [48].

### 5.8. Actinobacillus pleuropneumoniae

Various studies differently assessed the duration of the MDA against *Actinobacillus pleuropneumoniae*. Based on the available data, we can assume that a specific MDA is present in piglets from 2 to 12 weeks of age [50,51,52]. Research from 2004 demonstrated that the presence of the MDA during vaccination did not interfere with this process [68]. On the other hand, another study showed that piglets with high MDA titres immunised at 6 and 10 weeks of age did not exhibit any active antibody response [52]. These results indicate that high levels of the MDA may interfere with vaccines against *Actinobacillus pleuropneumoniae*. It can thus be assumed that the vaccination of piglets should not be conducted during the first weeks of life, as vaccine efficacy may be decreased; the administration of second and third doses of the vaccine later, at the ages of 10 and 14 weeks, may provide better vaccine efficacy in the presence of the MDA [52].

### 5.9. Erysipelotrix rhusiopathiae

The MDA against *Erysipelotrix rhusiopathiae* can remain above the positive cut-off level until eight weeks of age [53]. High MDA titres during vaccination negatively influence the effectiveness of this process; MDAs can affect the development of both the antibody as well as the cell-mediated immune response [53]. Seroconversion was noted when MDA-positive piglets were vaccinated at 8 and 10 weeks of age, but not at 6 weeks of age; nevertheless, this humoral response did not last long [53]. Two weeks post-vaccination of the MDA-positive piglets, the T-cell response was observed in 100% of piglets immunised at 8 and 10 weeks of age but only in 25% of piglets vaccinated at 6 weeks of age [53]. However, one study has shown that a vaccine prepared from an NaOH-extracted *Erysipelotrix rhusiopathiae* antigen can induce protective immunity even in the presence of MDAs [69].

### 5.10. Escherichia coli

To our knowledge, the data concerning the interference of the MDA against *E. coli* with the vaccination of piglets are scarce. It is known that the intramuscular vaccination of MDA-positive piglets may result in the induction of F4-specific antibodies [70]. Nguyen et al. (2015) conducted a study in which an assessment of MDI influence on the induction of the systemic immune response as a result of the oral vaccination of piglets was performed. This experiment showed that an active immune response could be achieved when MDA-positive piglets were immunised with oral vaccines. Moreover, MDI was considered to increase the secondary systemic immune response [70].

### 5.11. Glässerella parasuis

*Glässerella parasuis* (*G. parasuis*), formerly known as *Haemophilus parasuis*, is an important pathogen of swine. It was described that the MDA against *G. parasuis* remained above the positive level until three weeks of age [54]. The data concerning the interference of MDA with these bacteria is not consistent. A study by Pomorska-Mól et al. (2011) exhibits that the presence of MDA may adversely influence the development of seroconversion and the duration of active post-vaccinal antibody response [54]. The strongest seroconversion was observed in the piglets that were vaccinated at four and seven weeks of age; in the piglets vaccinated earlier (at one and four weeks of age and at two and five weeks of age), only minimal seroconversion was noted, and antibodies did not achieve positive values [54]. The later study, however, obtained different results [55]. It was shown that immunised piglets (at one and three weeks of age) born to vaccinated sows displayed significantly higher levels of specific IgG antibodies, lymphocyte proliferation as well as interferon-γ-secreting cells in comparison to vaccinated piglets born to non-vaccinated sows [55]; that may imply that no effective interference occurred. Nevertheless, it is worth mentioning that all vaccinated newborn piglets also obtained a prophylactic antibiotic injection [55].

### 5.12. Mycoplasma hyopneumonaie

One study assessed the duration of the MDA against *Mycoplasma hyopneumoniae* at 4–9 weeks of age [56]; however, another one showed that a decline in the MDA may even occur at the age of two weeks [57]. It was also observed that the percentage of seropositive immunised piglets (twice, at one and three weeks of age) seemed to decrease in the group of piglets born to seropositive sows [57]. This statement is in accordance with some later studies [23,58,59]. For example, Hodgins et al. (2002) demonstrated that piglets with high levels of the MDA during vaccination did not exhibit any growth in antibody titre after vaccine administration [58]. On the other hand, moderate levels of the MDA in two-week-old piglets did not disturb the development of a post-vaccinal active immune response in this group of animals [58]. Other researchers reported that interference between the MDA and vaccine occurred when MDA-positive piglets were immunised at one and four weeks of age; in contrast, piglets vaccinated at four and eight weeks of age exhibited a stronger immune reaction [59]. Similarly, Bandrick et al. (2014) observed that MDI-positive piglets vaccinated at one week of age could not develop antibody-mediated immunity [23]. Nevertheless, primary and secondary cellular-mediated immune responses were noted in these piglets. Thus, it can be assumed that MDI does not interfere with post-vaccinal cellular-mediated immunity [23]. However, not all researchers agree that MDAs interfere with developing an active immune response. Martelli et al. (2006) reported that the presence of MDAs during the vaccination of piglets at one week of age does not influence the vaccine-induced priming nor anamnestic response [60]. Another study showed that vaccination of MDA-positive piglets younger than one week of age could be effective; a significantly lower percentage of lung lesions and lower bacterial counts (in bronchial swabs and lung tissue) was observed [71]. Moreover, significantly higher antibody titres were noted in all immunised (MDA-positive and -negative) piglets compared to non-vaccinated animals [71]. Additionally, Reynolds et al. (2009) demonstrated that early immunisation at approximately one week of age conferred a long-lasting immunity in both seronegative and seropositive piglets [72]. Moreover, vaccinated piglets belonging to both groups were characterised by a significant decrease in the extent of lung lesions following virulent *Mycoplasma hyopneumoniae* exposure at later stages of life [72].

## 6. Infections with Immunosuppressive Pathogens

Several infectious agents, such as PRRSV or PCV-2, can cause immunosuppression in pigs. It can be thus assumed that infection with these pathogens can impair vaccine efficiency. It is well known that PRRSV negatively influences the host’s immune response. Infection with PRRSV can result in weak innate immunity and delayed and inefficient specific immunity [73]; for example, cytolysis, the inhibition of phagocytosis and antigen presentation or a cytokine pattern shift along with decreased immunostimulatory functions were documented [73]. Adverse immunomodulation observed in the course of infection with PRRSV is considered to be caused by the induction of interleukin (IL) 10, which is known for its immunosuppressive property [73]. Moreover, impaired local lung defence mechanisms in PRRSV infections contribute to developing secondary bacterial infections. Thus, several studies have evaluated whether PRRSV infection interferes with the efficacy of various vaccines [74,75,76,77,78]. It has been shown that PRRSV infection affects the development of the immune response following PRV vaccination [74]. The results of this study indicate that PRRSV infection does not influence PRV vaccine efficacy [74]. Admittedly, the time course of the T-cell response was adversely affected in PRRSV-infected pigs; however, the cytolytic response and the development of antibody titres were equal in both vaccinated groups (PRSSV-infected as well as negative control). Moreover, PRV was absent in both groups of pigs following the challenge [74]. Sinha et al. (2010) showed that PRRSV infection during vaccination against PCV-2 did not negatively affect vaccine effectiveness [78]. Regardless of the presence of PRRSV infection, vaccinated pigs developed specific antibodies [78]. Protection against PCV-2 infection was confirmed, as PCV-2 viremia and PCV-2 antigen prevalence in lymphoid tissue following PCV-2 challenge was reduced in vaccinated pigs compared to non-vaccinated ones [78]. Results of other studies, however, have indicated that PRRSV infection during vaccination can adversely influence the efficacy of this process [75,76,77]. For example, it has been found that PRRSV-infected pigs, vaccinated against CSF, were characterised by a significantly inhibited antibody response following immunisation compared to the control group [75]. Suradthat et al. (2005) demonstrated that the immunisation of pigs against CSF during the acute phase of PRRS may lead to the complete failure of this process due to the suppression of host immune response mechanisms [76]. In the mentioned study, pigs vaccinated against CSF during PRRSV infection exhibited clinical signs of disease following the CSFV challenge; however, the observed symptoms were milder than in non-vaccinated pigs. Nevertheless, most of the pigs from this group subsequently died, with a survival rate statistically indifferent from groups of non-vaccinated, CSFV-challenged pigs. Furthermore, pigs from this group were characterised by high CSFV titres in sera and other tissues; the pathological changes observed among this group were similar to those noted in non-vaccinated pigs. Moreover, PRRSV-positive pigs exhibited significantly lower anti-CSFV-neutralising titres following vaccination than negative ones during immunisation. No significant increase in antibody response in reaction to CSFV exposure was demonstrated in this group [76]. It has also been documented that the presence of PRRSV may significantly reduce SIV vaccine effectiveness [77]. In pigs that were infected with PRRSV at the time of vaccination, the more severe clinical signs, such as increased SIV shedding and elevated levels of macro- and microscopic lesions following SIV exposure, were observed compared to pigs that were PRRSV-negative during vaccine administration [77]. Interestingly, serum HI antibody response was not affected by the presence of PRRSV infection at vaccination [77]. PRRSV infection is also believed to reduce *Mycoplasma hyopneumoniae* bacterin vaccination effectiveness [79].

Another pathogen of which an infection influences the host lymphatic system is PCV-2, which is involved in developing PCV-2-associated disease (PCVAD). PCV-2 leading to lymphocyte depletion is considered a hallmark lesion of this infection [80]. The reduction in the number of lymphocytes results from apoptosis of dividing cells, macrophages and B-lymphocytes induced by PCV-2 [81]. Several studies indicated that PCVAD-affected pigs were characterised by a decreased lymphocyte number of various subsets (CD3+, CD4+, CD8+, CD4+CD8+) compared to control groups. It was assumed that this phenomenon could impair the host immune system and lead to immunosuppression [80,82]. PCV-2 also influences host innate, non-adaptive immunity; it can modulate the activity of dendritic cells and inhibit the activity of various innate immune cells, such as macrophages or NK cells [82]. The importance of the impact of PCV-2 on the factors of innate immunity results from the fact that dendritic cells, as well as macrophages, are responsible for the presentation of antigens to T-lymphocytes, starting with the adaptive immune response [83]. It is reasonable, thus, to suspect that PCV-2 infection can interfere with vaccine efficacy. Opriessnig et al. (2006) documented that PCV-2 infection during vaccination against PRRSV negatively influenced the development of immunity following vaccine administration [80]. In the mentioned study, however, no differences in IgG antibody response and the presence of PRRSV-NA were observed between pigs vaccinated against PRRSV with or without previous PCV-2 infection [80]. Nevertheless, an increase in PRRSV-induced micro- and macroscopic lung lesions following PRRSV exposure was observed in pigs subclinically infected with PCV-2 before PRRSV infection; these pigs also displayed a higher incidence of PRRSV antigens in the lungs [80]. Moreover, a significantly lower average daily weight gain (ADWG) was observed in pigs that were PCV-2-infected before PRRSV vaccination and challenge, similar to that observed in the non-vaccinated PRRSV-challenged group [80]. The role discovered of 2016 PCV-3, which is spread worldwide, in pigs’ health is still not fully recognised; however, it is considered that PCV-3 may contribute to the development of several pathological conditions, such as porcine dermatitis and nephropathy syndrome (PDNS) [83,84,85]. The emergence of novel PCV-3 yields questions about whether PCV-3 infection may influence PCV-2 vaccine efficacy. Based on the study performed by Woźniak et al. (2019), we can assume, however, that circulation of PCV-3 has no impact on PCV-2 vaccination effectiveness [80].

Another etiological agent that is not considered an important porcine pathogen but can cause immunosuppression is *Trypanosoma evansi* [86]. It was described that pigs infected with *Trypanosoma evansi* were characterised by a significantly decreased antibody response to the CSF vaccine and test antigen compared to the non-infected group [86]. Pigs immunised against CSF in the presence of *Trypanosoma evansi* displayed high fever and leukopenia following the CSF challenge. Thus, protection was insufficient [86]. The authors concluded that heterologous vaccines could be inefficient in providing protective immunity in the presence of *Trypanosoma evansi* infection [86].

## 7. Antibiotic Usage

Antibiotics are commonly used in the pig industry. It has been documented that except for their antimicrobial properties, antibiotics can also influence the immune system by a.o. modulation of cytokine secretion, antibody production or T-cell proliferation [87]. As in the field, antibiotics are sometimes administered to pigs simultaneously with vaccines; it is reasonable to suspect that they can somehow affect pig vaccination efficacy. Several studies evaluated this phenomenon using various antibiotics (Table 2). Doxycycline belongs to the tetracycline family and is commonly used for infections caused by Gram-positive and Gram-negative bacteria [88]. Numerous studies proved that doxycycline usage could adversely affect immune response. Considering the immunomodulating properties and common usage of doxycycline in pig production, often simultaneously with vaccination, the concerns regarding its interference with vaccine efficacy in pigs seem reasonable. A study by Pomorska-Mól et al. (2014) indicates that oral doxycycline therapy can modulate the cell-mediated post-vaccinal immune response in pigs following PRV immunisation [88]. Vaccinated pigs treated with doxycycline displayed a significant decrease in IFN-γ production compared to vaccinated pigs deprived of doxycycline treatment following PRV stimulation. Moreover, a significant decrease in the stimulation index values was observed in these pigs after PRV restimulation. Two weeks following the second dose of the vaccine administration, doxycycline-treated pigs also exhibited a lower percentage and absolute number of double-positive CD4+CD8+ lymphocytes than pigs that were not obtaining antibiotics; nevertheless, they still had a higher absolute count of double-positive cells than non-vaccinated pigs. Despite that doxycycline treatment impaired the post-vaccinal cell-mediated immune response, no significant impact on the humoral response was noted [88]. In another study, the influence of doxycycline on the humoral immune response caused by *Erysipelothrix rhusiopathiae* vaccination was assessed, and its results proved that simultaneous doxycycline treatment and vaccination might result in a decrease in the production of specific antibodies [87]. Another antibiotic commonly used in the pig industry and known for its immunomodulating properties is enrofloxacin, which belongs to fluoroquinolones [89]. According to the results of another study, simultaneous vaccination against PRV and treatment with enrofloxacin may alter the immune response to the vaccine. The administration of therapeutic doses of enrofloxacin influenced both the humoral and cellular post-vaccinal immune response against PRV [90]. Enrofloxacin-treated pigs displayed delayed seroconversion as compared to pigs deprived of treatment. Furthermore, this group exhibited a significant decrease in IFN-γ production following the PRV challenge. Upon PRV restimulation, significantly lower values of stimulation index were noted in pigs treated with enrofloxacin than in those non-treated. Enrofloxacin also affected the secretion of other cytokines, such as IL-6, IL-10 and tumour necrosis factor (TNF) α by peripheral blood mononuclear cells (PBMC) following stimulation [89]. Humoral and cellular immunity following PRV and H3N2 SIV immunisation was modulated in pigs vaccinated during ceftiofur hydrochloride treatment [90]. PRV vaccination combined with ceftiofur treatment resulted in delayed development of humoral response; however, significant differences between PRV-vaccinated pigs with or without ceftiofur treatment were only observed until booster administration. PRV-vaccinated, ceftiofur-treated pigs also displayed a significant decrease in IFN-γ production. Furthermore, this group reached significantly lower stimulation index values following PRV restimulation. Ceftiofur treatment combined with SIV vaccination resulted in a marked reduction in the anti-HA3 antibodies; pigs vaccinated during antibiotic therapy exhibited a significantly lower HI titre than those not treated with ceftiofur. Moreover, six weeks after the second dose of the SIV vaccine, only 7 out of 15 ceftiofur-treated pigs had HI titres that provided immunity against influenza; meanwhile, in the group with no antibiotic therapy, all the pigs achieved protective levels of HI. However, no influence on the cellular immunity against SIV was observed; any significant differences in IFN-γ secretion and regarding stimulation index were found between the ceftiofur-positive and -negative vaccinated groups [90]. Another study has shown that the vaccination of pigs with inactivated *Erysipelothrix rhusiopathiae* vaccine at the time of ceftiofur treatment may significantly decrease specific antibody production [87]. Similar effects were observed for tiamulin. In contrast, interestingly, simultaneous vaccination and treatment with amoxicillin or tulathromycin resulted in the enhancement of antibody development [87]. The exact mechanism by which antibiotics influence the post-vaccinal immune response is not clarified yet; however, it is suspected that it may be due to the effects antibiotics exert on various cytokine secretions [87,88].

## 8. Dietary Factors—Mycotoxins

Mycotoxins are secondary metabolites of fungi, mainly belonging to *Aspergillus, Fusarium* and *Penicillium* genus, that have a toxic impact on animal and human health. Various mycotoxins may induce different effects; for example, they can impair the functions of particular organs or cause carcinogenic or teratogenic effects [91]. Nevertheless, the contamination level of pigs’ feed with mycotoxins is frequently insufficient to induce any clinical signs of disease; however, it may be efficient in impairing production parameters that subsequently lead to economic losses or in the induction of immunosuppression [91]. Immunosuppression always poses a threat of inadequate vaccine efficacy. The possible interference of various mycotoxins with vaccines was assessed in multiple studies. One of the mycotoxins harmful to the immune system is aflatoxin, which exhibits immunosuppressive properties and can adversely affect the innate and specific immune response [91,92]. In pigs fed with aflatoxin-contaminated feed, an interference with the development of specific immunity following *Erysipelotrix rhusiopathiae* bacterin vaccination was documented; aflatoxin-exposed individuals were more susceptible to infection due to *Erysipelotrix rhusiopathiae* during post-vaccinal challenge in comparison to immunised ones that were fed with no aflatoxin addition [93]. In pigs immunised with the ovalbumin model antigen, the presence of AFB1 in the pigs’ diet had no major impact on humoral immunity; the concentrations of IgG, IgM, IgA, and anti-ovalbumin IgG were not significantly modulated [92]. AFB1 exposure did not impair the lymphocyte mitogenic response; nevertheless, it delayed and reduced their specific proliferation following vaccination. This phenomenon may implicate an interference with lymphocyte activation due to AFB1 feed contamination, which can further contribute to vaccination failures [92]. Deoxynivalenol (DON), which belongs to type B trichothecenes, is a mycotoxin that can display both immunostimulatory and immunosuppressing properties, depending on its dose and frequency of exposure [94]. Pigs are among the animals that are highly sensitive to DON; the ingestion of DON may influence their systemic immune response [95]. Two independent studies investigated the impact of DON consumption on pigs’ post-vaccinal immune response [95,96]. It has been reported that the consumption of DON modulated the pigs’ immune response following ovalbumin vaccination; an increase in the ovalbumin-specific IgA and IgG in pigs fed with DON addition was observed [96]. Lessard et al. (2015) also observed an increase in IgG levels in pigs vaccinated with ovalbumin exposed to DON compared to the control group [95]. Even though DON did not modulate lymphocyte proliferation following mitogenic stimulation, it exhibited a biphasic impact on this process after antigenic stimulation: up-regulation at first and down-regulation later [96]. Moreover, the levels of mRNA expression of TGF-β and IFN-γ in the mesenteric lymph nodes were lower compared to control pigs. Interestingly, an up-regulated expression of INF- γ and IL8 in DON-exposed pigs has also been observed following ovalbumin vaccination [95]. These findings indicate a modulation of the post-vaccinal immune response by DON, which may potentially result in breakdowns of post-vaccine immunity [95,96]. Subsequently, it was demonstrated that DON could suppress vaccination efficacy against PRRSV, as the ingestion of this mycotoxin by immunised pigs resulted in strongly impaired viral replication; post-vaccination viremia occurred only in 17% of pigs that ingested 3.5 mg of DON/kg and in 50% of pigs receiving 2.5 mg of DON/kg [97]. Pigs fed with DON-contaminated feed developed PRRSV-specific antibodies only if they were viraemic [97]. Another mycotoxin that may impair immune response is T-2 toxin, which belongs to the type A trichothecenes; an adverse impact on the lymphatic system results from its haematotoxicity [98]. Meissonnier et al. (2008) conducted a study in which the toxic influence of this mycotoxin on the humoral immune response was documented. The immunisation of pigs that ingested feed contaminated with T-2 toxin with ovalbumin led to a decrease in anti-ovalbumin antibody levels with no significant impact on specific lymphocyte proliferation [98]. Some studies have also proved that fumonisin B_1_ (FB_1_) has immunosuppressive properties in pigs [99]. In the experiment by Taranu et al. (2005), a decrease in specific antibody titres following *Mycoplasma agalactiae* vaccination was observed in pigs prolongedly exposed to FB_1_ in contaminated feed; contrarily, no impact on the serum concentration of IgG, IgM, or IgA was observed [100]. Moreover, FB_1_ negatively affected the cytokine profile, as the expression of IL-4 mRNA by porcine whole-blood cells was diminished [100]. Interestingly, the subsequent study demonstrated that the consumption of feed with the FB_1_ addition caused a significant decrease in specific antibody levels following *Mycoplasma agalactiae* vaccination in male individuals; meanwhile, in females, no impact was observed [99]. Moreover, males also exhibited diminished IL-10 mRNA expression levels [99]. The obtained results indicated that the degree of immunosuppression caused by FB1 exposure might strongly depend on sex [99]. According to Stoev et al. (2012), not only FB_1_ but also ochratoxin A, as well as their combinations, can affect the humoral immune response, as a strong decline in antibody levels against Aujeszky’s disease at 21 and 35 days following vaccination was noted in pigs that were exposed to these mycotoxins [101].

## 9. Conclusions

Multiple antibiotic usage restrictions introduced in numerous countries have led to a significant increase in the importance of vaccination. Proper immunisation protocols can protect pigs against various infectious diseases and, therefore, economic losses. However, sometimes vaccinations do not yield the expected effects and result in failures. One of the reasons for these failures can be a lack of awareness that vaccination is a more complex issue than it can appear to be at first sight. Pigs during their life are exposed to many various factors that may influence their immune systems. Thus, it is reasonable to assume that these factors can also affect their immune response following vaccination. In the present study, we reviewed seven groups of elements, and in the majority of the quoted reports, their influence on vaccination efficacy was adverse. Some of these factors, such as the influence of MDAs, are well-known and deeply examined. Therefore, vaccination failures for this reason are relatively rare; the immunisation scheme regarding such factors results from their threats to the efficacy of the process. On the other hand, a greater group of agents, such as stress or the microbiome, has been poorly investigated with few or even a single number of reports, which is insufficient to provide a complete understanding of their impact on vaccination failures. As a result, such factors need to be considered during the planning of the vaccination schedule since they can affect vaccine efficacy, raising questions among animal owners about their usefulness as an effective alternative for antibiotics. Therefore, to maximise the profits that can arise from pig immunisation, an intensification of research involving the factors that are likely to interfere with vaccines but are poorly or not completely investigated in this aspect is required. Moreover, a propagation of awareness about various agents that can result in vaccine failure among animal owners and veterinarians seems crucial. Successful vaccination protocols are more complex than they appear due to multiple factors that may interact with post-vaccinal immunity; a holistic approach that considers all these agents is thus required to achieve adequate vaccine efficacy.

## Figures and Tables

**Table 1:** Duration of MDAs against various pathogens and their influence on the vaccine-induced immune response.

Pathogen	Duration of MDA	Type of Vaccine	Interference with Humoral Immunity	Interference with Cellular Immunity	References
Viral agents					
CSFV	7–10 weeks	Attenuated live Chinese strain virus vaccine	Yes	NE ^1^	[25,26,27,28]
		Lapinized Chinese-strain CSF vaccine	Yes	Yes	
		E2 subunit vaccine	Yes	NE	
FMDV	2 months	0_1_BFS 1860	Yes	NE	[22,29,30]
		Inactivated O/TWN/97	Yes	NE	
		Inactivated A TUR/14/98	Yes	NE	
PCV-2	2–15 weeks	Killed PCV1-2 chimeric vaccine (Suvaxyn PCV-2One Dose)	Yes	NE	[31,32,33,34,35,36,37]
		PCV-2a-based subunit vaccine (Porcilis PCV)	Yes	NE	
		Inactivated PCV-2 vaccine (Circovac)	Yes	NE	
		Batch no A021A01(Porcilis PCV)	Yes	NE	
		Inactivated subunit vaccine (Porcilis PCV)	Yes	NE	
		PCV-2a-based subunit vaccine (Porcilis PCV)	Yes	NE	
		Batch number 309-762B (Ingelvac Circoflex)	Yes	NE	
PPV	6 months	Probably inactivated	No	NE	[38,39]
		Inactivated vaccine	Yes	NE	
PRV	3–11 weeks	gE-deleted vaccine	Yes	Yes	[17,40,41,42]
		Inactivated strain NIA-4 (Auskimune IN)	Yes	NE	
		Inactivated (Nobivac Aujeszky)	Yes	NE	
		gE-deleted vaccine	Yes	NE	
		Attenuated Bartha strain	Yes	NE	
PRRSV	2–11 weeks	Modified live vaccine (Porcilis PRRS)	Yes	Yes	[24,43,44,45]
		Modified live vaccine (Porcilis PRRS)	Yes	NE	
SIV	9–16 weeks	Bivalent inactivated whole SIV H1N1 (A/sw/Ollost/84) and H3N2 (A/Port Chalmers/1/73)	Yes	NE	[18,46,47,48,49]
		Bivalent SIV H1N1, H3N2 (FluSure)	Yes	Yes	
		H1N1pdm09 whole inactivated virus	Yes	NE	
		Quadrivalent H1N1-γ, H1N2-δ1, H1N1-δ2 and H3N2-IV vaccine (Flusure XP)	Yes	NE	
Bacterial agents					
*Actinobacillus pleuropneumoniae*	2–12 weeks	Subunit vaccine containing OMP and ApxI, ApxII, and ApxIII toxins (Porcilis APP)	Yes	NE	[50,51,52]
*Erysipelotrix rhusiopathiae*	8 weeks	Live vaccine (Inglevac)	Yes	Yes	[53]
		Live vaccine	Yes	NE	
*Glässerella parasuis*	3–5 weeks	Inactivated vaccine	Yes	NE	[54,55]
		Inactivated vaccine (Suvaxyn)	Yes	Yes	
*Mycoplasma hyopneumoniae*	2–9 weeks	Inactivated vaccine (Stellamune Mycoplasma)	Yes	NE	[23,56,57,58,59,60]
		*M. hyopneumoniae* inactivated bacterin	Yes	NE	
		Inactivated vaccine (Hyoresp)	Yes	NE	
		Bactrin (Respisure-One)	Yes	No	

^1^ NE = Not evaluated.

**Table 2:** Influence of concurrent antibiotic therapy and vaccination on the post-vaccinal immune response.

Antibiotic	Dose	Route of Administration	Vaccine	Influence on Humoral Immunity	Influence on Cellular Immunity	References
Doxycycline	12.5 mg/kg/day	PO ^1^	Live-attenuated gE-deleted PRV vaccine	No	Yes	[88]
	12.5 mg/kg/day	PO	Inactivated Erysipelothrix rhusiopathiae vaccine	Yes	NE ^2^	[87]
Enrofloxacin	1 ml/kg/day	IM ^3^	Live-attenuated gE-deleted PRV vaccine	Yes	Yes	[89]
Ceftiofur	3 mg/kg/day	IM	Live-attenuated gE-deleted PRV vaccine	Yes	Yes	[90]
	3 mg/kg/day	IM	Inactivated SIV vaccine	Yes	No	[90]
	3 mg/kg/day	IM	Inactivated Erysipelothrix rhusiopathiae vaccine	Yes	NE	[87]
Tiamulin	12 mg/kg/day	IM	Inactivated Erysipelothrix rhusiopathiae vaccine	Yes	NE	[87]
Amoxicillin	15 mg/kg/day	IM	Inactivated Erysipelothrix rhusiopathiae vaccine	Yes	NE	[87]
Tulathromycin	2.5 mg/kg/day	IM	Inactivated *Erysipelothrix rhusiopathiae* vaccine	Yes	NE	[87]

^1^ PO = per os, ^2^ NE = not evaluated, ^3^ IM = intramuscular.

## Data Availability

Data is contained within the article.
